# Use of Molecular Imaging Markers of Glycolysis, Hypoxia and Proliferation (^18^F-FDG, ^64^Cu-ATSM and ^18^F-FLT) in a Dog with Fibrosarcoma: The Importance of Individualized Treatment Planning and Monitoring

**DOI:** 10.3390/diagnostics5030372

**Published:** 2015-09-11

**Authors:** Kamilla Westarp Zornhagen, Malene M. Clausen, Anders E. Hansen, Ian Law, Fintan J. McEvoy, Svend A. Engelholm, Andreas Kjær, Annemarie T. Kristensen

**Affiliations:** 1 Department of Veterinary Clinical and Animal Sciences, Faculty of Health and Medical Sciences, University of Copenhagen, Dyrlaegevej 16, DK-1870 Frederiksberg C, Denmark; E-Mails: fme@sund.ku.dk (F.J.M); atk@sund.ku.dk (A.T.K.); 2 Department of Clinical Physiology, Nuclear Medicine & PET and Cluster for Molecular Imaging, Rigshospitalet and University of Copenhagen, Blegdamsvej 3B, DK-2200 Copenhagen N, Denmark; E-Mails: mmc@sund.ku.dk (M.M.C.); ilaw@pet.rh.dk (I.L.); akjaer@sund.ku.dk (A.K.); 3 Department of Radiation Oncology, Rigshospitalet, University of Copenhagen, Blegdamsvej 9, DK-2200 Copenhagen N, Denmark; E-Mail: svend.aage.engelholm@regionh.dk; 4 Department of Micro- and Nanotechnology, Technical University of Denmark, Ørsteds Plads, Building 345E, DK-2800 Kgs. Lyngby, Denmark; E-Mail: aeha@sund.ku.dk

**Keywords:** ^18^F-FDG, ^18^F-FLT, ^64^Cu-ATSM, positron emission tomography, PET/CT, individualized response monitoring, canine fibrosarcoma

## Abstract

Glycolysis, hypoxia, and proliferation are important factors in the tumor microenvironment contributing to treatment-resistant aggressiveness. Imaging these factors using combined functional positron emission tomography and computed tomography can potentially guide diagnosis and management of cancer patients. A dog with fibrosarcoma was imaged using ^18^F-FDG, ^64^Cu-ATSM, and ^18^F-FLT before, during, and after 10 fractions of 4.5 Gy radiotherapy. Uptake of all tracers decreased during treatment. Fluctuations in ^18^F-FDG and ^18^F-FLT PET uptakes and a heterogeneous spatial distribution of the three tracers were seen. Tracer distributions partially overlapped. It appears that each tracer provides distinct information about tumor heterogeneity and treatment response.

## 1. Case Presentation

A nine-year-old spayed, female, mixed-breed dog weighing 25 kg was referred to the oncology service at the University Hospital for Companion Animals, Department of Veterinary Clinical and Animal Sciences, University of Copenhagen, Denmark, with a suspected bone tumor in the proximal left tibia. The initial oncological work-up showed normal blood profiles and no evidence of metastases on three-view thoracic radiography or on fine needle aspirate from the left popliteal lymph node. Investigations also included CT and bone biopsies. The left proximal tibia showed both radiolucent and radiopaque areas, with most pronounced changes being found in the midplane and laterally. The lateral fabella showed irregular contours. There was no evidence of involvement of the stifle joint or on the thoracic CT of distant metastasis. Histopathology findings included infiltration of the marrow spaces by collagen and proliferating spindle cells, some of which showed minimal atypia and rare mitoses. Most sections showed no significant mitotic activity or atypia. Sarcoma was given as a histopathological diagnosis with a comment that the growth pattern and morphology was most consistent with a fibrosarcoma.

Curative intent left hind limb amputation was recommended but declined. Radiotherapy (10 fractions of 4.5 Gy) was then offered, and as part of on-going research of PET tracers in cancer patients, it was possible to also offer PET/CT scan sequences for staging purposes and to monitor therapeutic response. The Ethics and Administrative Committee at the Department of Veterinary Clinical and Animal Sciences, Faculty of Health and Medical Sciences, University of Copenhagen, Denmark, approved the pilot study protocol and owners provided written consent.

## 2. PET/CT Imaging during Radiotherapy—Methods

PET/CT scans with the glucose analogue, 2-deoxy-2-[^18^F]fluoro-d-glucose (^18^F-FDG); the cell proliferation PET tracer 3′-deoxy-3′-[^18^F]fluorothymidine (^18^F-FLT) [1,2]; and one promising hypoxia PET tracer ^64^Cu-diacetyl-bis(N^4^-methylthiosemicarbazone) (^64^Cu-ATSM) [3,4] were performed on consecutive days one week prior to the first radiotherapy fraction, after five and 10 fractions of radiotherapy, and finally at 10 weeks after the end of treatment.

The initial ^18^F-FDG PET/CT scan data was used for the radiotherapy plan. The gross tumor volume (GTV) was delineated on the CT by cooperation between a veterinarian and an experienced radiologist. Furthermore, a specialist in nuclear medicine helped in incorporating the evaluation of the ^18^F-FDG PET in the GTV.

All scans were performed on the same combined PET/CT scanner (Biograph40, Siemens, Munich, Germany) consisting of a 40-slice CT scanner and a high-resolution PET scanner. CT parameters were: 120 kV, 170 mAs, pitch 1.2, collimation 24 × 1.2 mm, slice thickness 3.0 mm, and a B30 kernel. PET scans were acquired using a three-dimensional (3D) acquisition mode and True X reconstruction (point spread function, three iterations, 21 subsets, Syngo MI. PET/CT 2008A, Siemens, Munich, Germany) and smoothed using a Gaussian filter having a full width at half maximum of 2 mm, and a matrix size of 336 × 336.

Radiotherapy was administered using a linear accelerator (Novalis Tx™, Varian Medical Systems, Inc., Palo Alto, CA, USA). Six MV photons were given as a conformal 3D field technique. ExacTrac X-ray 6D (BrainLab, Feldkirchen, Germany) and cone beam CT were used to ensure precise and uniform positioning.

The canine patient fasted for at least 12 h prior to all PET/CT scans and radiotherapy. Anesthesia during the procedures was induced with propofol (4 mg/kg, B. Braun Medical A/S, Frederiksberg, Denmark) after pre-medication with methadone (0.2–0.3 mg/kg i.m., Comfortan Vet 10 mg/mL, Dechra Veterinary Products A/S, Uldum, Denmark). Anesthesia was maintained using the continuous rate infusion of propofol (15–25 mg/kg/h) with 100% oxygen via an endo-tracheal tube. Heart rate, oxygen saturation, blood pressure, and CO_2_ concentrations were monitored throughout the anesthesia. During radiotherapy and PET/CT scans, the canine patient was positioned in a vacuum-fixed pillow to achieve precise and uniform positioning. Isocentric lasers and markers of copper wire were used to re-establish the correct patient position between individual scans and treatments.

Before each ^18^F-FDG PET/CT scan, blood glucose measurements confirmed normal blood glucose levels. ^18^F-FDG from daily routine productions for clinical use and ^18^F-FLT produced as previously described [5] were produced at the Cyclotron Unit, Department of Clinical Physiology, Nuclear Medicine and PET, Copenhagen University Hospital, Denmark. ^64^Cu-ATSM was purchased from Hevesy Laboratory, DTU Campus Risø, Denmark.

All tracers were injected as an intravenous bolus of approximately 7.2 MBq/kg with slight variations between tracers and scans. ^18^F-FDG and ^18^F-FLT were injected approximately 1 h (range 56–95 min, median 69.5 min for ^18^F-FDG; range 60–86 min, median 76 min for ^18^F-FLT) prior to PET/CT scanning while ^64^Cu-ATSM was administered about 6.5 h (range 381–422 min, median 406.5 min) prior to scanning. Both ^18^F-FDG and ^18^F-FLT PET/CT scans were performed as 10-min single-field-of-view static scans of the tumor area, while ^64^Cu-ATSM PET/CT scans were executed as 20-min single-field-of-view scans. The first ^18^F-FDG PET/CT scan prior to radiotherapy planning furthermore included a 2-min five-fields-of-view full body PET scan to evaluate for metastasis. No metastases were found on the combined ^18^F-FDG PET/CT images.

Images from the same scan series were manually co-registered according to the CT images. PET uptake was quantified using the standardized uptake value (SUV), which is a unitless semi-quantitative measure that accounts for the injected dose and the body weight [6,7]:

*SUV* = Activity in tumor region (kBq/mL)/(Injected tracer activity (MBq)/body weight (kg)) (1)

Using the ^18^F-FDG PET/CT scan and 3D iso-contouring in TrueD (Syngo, Multi Modality Workplace VE40A, Siemens, Munich, Germany), a tumor volume defined by a SUV cut-off on 2.5 (^18^F-FDG SUV 2.5 volume) was delineated for each scan series (meaning the iso-contour-selected region where the ^18^F-FDG PET uptake is 2.5 SUV). These iso-contoured regions were applied as volumes of interest (VOIs) to the corresponding ^64^Cu-ATSM and ^18^F-FLT PET/CT images. Tumor maximum and mean SUVs (SUV_max_ and SUV_mean_) of all tracers were subsequently determined in the ^18^F-FDG SUV 2.5 volumes/VOIs. Furthermore, a SUV_max_-region defined as 90% of the maximum SUV was delineated for each tracer and scan.

The SUV cut-off on 2.5 was chosen since it has been used to discern between benign and malignant lesions when evaluating, for instance, lung and adrenal masses [8,9]. Additionally, in soft tissue lesions, ^18^F-FDG PET is good at differentiating between benign and malignant [10]. Furthermore, in studies of Ewing sarcomas and extremity osteosarcomas, a ^18^F-FDG SUV value under 2.5 during scanning after neoadjuvant chemotherapy was predictive of progression-free survival [11,12].

## 3. Results and Outcome

**Figure 1:**
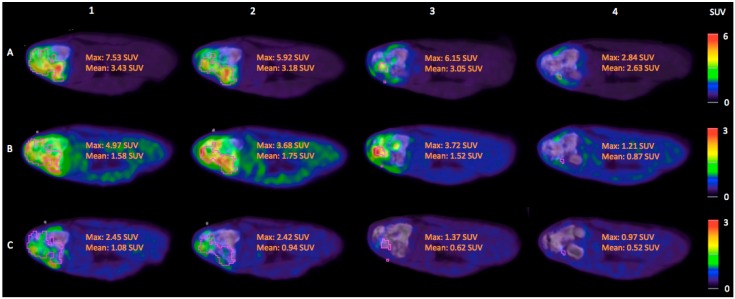
PET/CT images of the tumor area in the left proximal tibia, transverse plane. Both uptake magnitude and distribution are different between the three tracers. *Column 1*: Before radiotherapy; *Column 2*: After 22.5 Gy of radiotherapy; *Column 3*: After 45 Gy of radiotherapy (at completion of radiotherapy); *Column 4*: 10 weeks after completing radiotherapy. *Row A*: ^18^F-FDG PET/CT; *Row B*: ^18^F-FLT PET/CT; *Row C*: ^64^Cu-ATSM PET/CT 6.5 h after intravenous injection. Pink angular lines demarcate 3D iso-contouring lines for the tumor volume of interest (VOI) defined by a standardized uptake value (SUV) cut-off at 2.5 for ^18^F-FDG (^18^F-FDG SUV 2.5 volume). First delineated on the ^18^F-FDG PET/CT images, these VOIs were subsequently applied to the corresponding ^18^F-FLT and ^64^Cu-ATSM PET/CT images. The maximum and mean SUV values for the respective tracers in these VOIs/^18^F-FDG SUV 2.5 volumes are given in orange text. ^18^F-FDG PET/CT images are in window levels of SUV 0–6, while ^18^F-FLT and ^64^Cu-ATSM PET/CT images are in window levels of SUV 0–3 as also indicated by the color scale bars.

**Figure 2:**
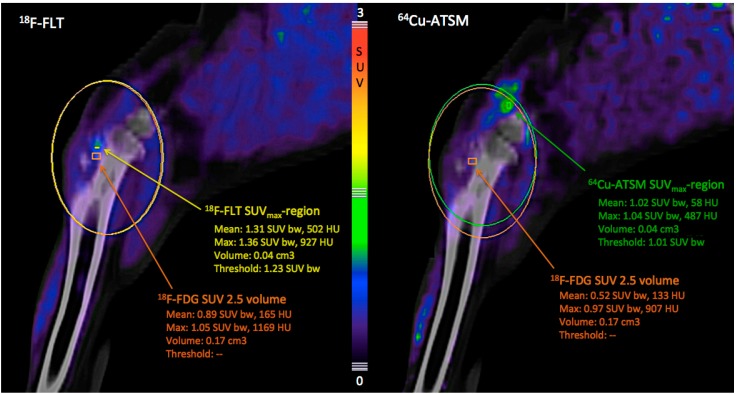
PET/CT images of the tumor area in the left proximal tibia, sagittal plane, 10 weeks after completion of radiotherapy. The SUV_max_-region for ^18^F-FLT (left picture) and ^64^Cu-ATSM (right picture) are localized outside the ^18^F-FDG SUV 2.5 volume. Orange rectangles demarcate 3D iso-contouring lines for the tumor volume of interest (VOI) defined by a standardized uptake value (SUV) cut-off at 2.5 for ^18^F-FDG (^18^F-FDG SUV 2.5 volume). The maximum and mean SUV values for the respective tracers in this VOI are given in orange text. The yellow rectangle demarcates 3D iso-contouring lines for the ^18^F-FLT SUV_max_ region. This is a region defined by 90% of the maximum SUV for ^18^F-FLT in the tumor area. The maximum and mean SUV values for ^18^F-FLT uptake in this SUV_max_-region are given in yellow text. The green rectangle demarcates 3D iso-contouring lines for the ^64^Cu-ATSM SUV_max_ region. This is a region defined by 90% of the maximum SUV for ^64^Cu-ATSM in the tumor area. The maximum and mean SUV values for ^64^Cu-ATSM uptake in this ^64^Cu-ATSM SUV_max_ region are given in green text.

Tumor uptake of ^64^Cu-ATSM decreased continuously within the volume defined by ^18^F-FDG SUV 2.5 during and after the course of radiotherapy, resulting in the reduction of ^64^Cu-ATSM SUV_max_ of >60% from the pre-treatment to the 10-week post-treatment scan (Figure 1). The same order of reduction from the pre-treatment to the 10-week post-radiotherapy scans was also found for ^18^F-FDG SUV_max_ and ^18^F-FLT SUV_max_, though their uptake displayed fluctuations during radiotherapy (Figure 1). Furthermore, it was seen that ^18^F-FLT and ^64^Cu-ATSM might add information not available from ^18^F-FDG PET/CT alone, since the SUV_max_-regions of these two tracers were not co-localized with the ^18^F-FDG SUV 2.5 volume (Figure 2). However, the scan at the end of radiotherapy showed a co-localized region of ^18^F-FLT and ^18^F-FDG uptake (Figure 1, column 3).

The hypo-fractionated radiotherapy was well tolerated, the formation of an acute superficial moist dermatitis in the radiation field being the only sign of acute radiotherapy injuries. During and post-treatment, the canine patient showed varying but reduced degrees of lameness on the left hind limb. Lameness commenced after the fourth radiotherapy fraction, but decreased in the third week post-radiotherapy. Lameness increased again approximately five weeks post-radiotherapy, but could be controlled therapeutically, and about three months post-radiotherapy, the lameness was mild and intermittent, but there was still soreness at full extension of the left stifle joint. Lameness was first managed using firocoxib (Previcox 227 mg, Merial Norden A/S, Hørsholm, Denmark) initiated at initial presentation, but due to renal complication, this was withdrawn 4.5 months post-radiotherapy, leading to increasing degrees of lameness and pain. Physiotherapy twice weekly and, later, daily pain treatment with meloxicam (Metacam 1.5 mg/mL; Boehringer Ingelheim DK A/S, Copenhagen, Denmark, 0.1 mg/kg) made the canine patient improve. At seven months post-radiotherapy, there was an acute deterioration with an acute lameness. A pathological tibial fracture was diagnosed and acute left hind limb amputation was recommended, but the owners decided to euthanize the canine patient.

## 4. Discussion

To the authors’ knowledge, this pilot study is one of the first to illustrate the simultaneous use of three different PET/CT tracers for non-invasive functional imaging of molecular changes in a canine cancer patient before, during, and after radiotherapy. ^18^F-FDG, ^18^F-FLT, and ^64^Cu-ATSM PET uptakes decreased initially during treatment, and from the pre-treatment scan to the post-treatment scan 10 weeks after completing therapy. The decrease in ^64^Cu-ATSM PET uptake was continuous during and after radiotherapy, indicating either tumor reoxygenation in areas of preserved ^18^F-FDG uptake signifying viable tumor tissue or necrosis, or non-functional tumor tissue in areas of reduced ^18^F-FDG uptake. The ^18^F-FDG and ^18^F-FLT PET uptakes showed some fluctuations during treatment. These variations in image signals may be a result of the differences in latency from tracer injection to imaging between the different scan series. However, the fluctuations in ^18^F-FLT PET uptakes may also indicate accelerated repopulation during therapy, while tissue remodeling and inflammation may explain the fluctuations in ^18^F-FDG PET uptakes. This is due to the fact that ^18^F-FDG as a marker of glycolytic activity is not tumor-specific, but is also taken up by, for instance, macrophages during inflammation [13]. Inflammation during tissue remodeling may also account for the reduced but continued uptake of ^18^F-FDG 10 weeks after completing therapy, even though incomplete tumor control cannot be ruled out. The co-localization and focal increase in ^18^F-FDG and ^18^F-FLT seen at scanning after the end of radiotherapy (Figure 1, column 3) might indicate that clones of cancer cells are repopulating the tumor area after the end of radiotherapy. As the image signal for both ^18^F-FDG and ^18^F-FLT is decreased at scanning 10 weeks after the end of therapy, perhaps an evaluation of the radiotherapy response in canine soft tissue sarcomas should not be done directly after completion of radiotherapy, but rather after waiting for some weeks.

The visual interpretation of the scan images revealed a heterogeneous spatial distribution of the three tracers, which to some extent was overlapping, though not identical. This indicates that additional information about tumor heterogeneity and treatment response is gained through multi-tracer imaging. The use of multiple tracers may also be beneficial for individualized treatment planning and to predict outcome, since more aspects of the tumor phenotype are elucidated. This is illustrated in a recent study which showed that ^64^Cu-ATSM and ^18^F-FDG provide different biological information to be taken into account when using dose painting for radiotherapy planning [14]. With dose painting, a non-uniform radiation dose distribution is prescribed to the target volume based on information from, for instance, PET scans on tumor areas that might be resistant and thus require a higher dose [15]. The aforementioned study focused on dose painting of hypoxic areas [14], since tumor hypoxia has an essential impact on the molecular mechanisms in solid cancers by up-regulating multiple genes resulting in an aggressive phenotype and treatment resistance [16]. Though targeting hypoxic tumor areas is of great interest in the field of radiotherapy planning, other phenotypically distinct tumor areas such as highly proliferative areas delineated by ^18^F-FLT PET may also be worth attending in future radiotherapy planning research.

Until recently, guidelines for evaluating solid tumors’ responses to treatment relied on anatomical imaging techniques such as CT and MRI (RECIST) [17,18]. Since ^18^F-FDG PET/CT has shown promising results for response monitoring during treatment in different human cancers [19], new guidelines using ^18^F-FDG PET/CT have emerged (PERCIST) [20]. Likewise, ^18^F-FLT has shown promising results for early response monitoring in human head and neck cancer [21,22]. Precisely the use of these two PET/CT tracers for response monitoring after radiotherapy in canine cancer patients was first published in a case report where ^18^F-FLT PET/CT in supplement to ^18^F-FDG revealed disease recurrence [23]. During the last two years, a couple of interesting canine studies have followed. A study of 10 canine patients with sinonasal cancers (seven adenocarcinomas, a chondrosarcoma, a osteosarcoma, and a squamous cell carcinoma) used pre-treatment ^18^F-FDG, ^18^F-FLT, and ^61^Cu-ATSM PET/CT scans and a single ^18^F-FDG PET/CT scan three months post-radiotherapy (50 Gy in 10 fractions) for spatially resolved regression analysis to investigate the impact of the pre-treatment scans in predicting the response to radiotherapy [24]. The results revealed that the pre-treatment ^18^F-FDG PET uptake was a significant positive predictor of three-month post-treatment ^18^F-FDG PET uptake, though with histopatological discrepancies, while baseline ^18^F-FLT and ^61^Cu-ATSM PET uptake did not contribute significantly to multivariate regression coefficients [24]. This study is, however, limited by the absence of follow-up PET/CT scans with ^18^F-FLT and ^61^Cu-ATSM. Members of the same research group in a later study compared the spatial correlation of ^18^F-FDG, ^18^F-FLT, and ^61^Cu-ATSM during PET/CT in a cohort of 22 canine patients with sinonasal cancers, eight of which were sarcomas [25]. They found a significantly greater overlap between the highest uptake volumes of the three tracers in carcinomas than in sarcomas, a finding comparable to the visual interpretations of our PET/CT images in a canine fibrosarcoma. A more recent study of the same canine cohort investigating the changes in ^18^F-FLT and ^61^Cu-ATSM PET uptakes between pre-treatment and mid-treatment PET/CT scans during hypo-fractionated radiotherapy showed a significant reduction in the ^61^Cu-ATSM uptake after three fractions of intensity-modulated radiation therapy (IMRT) (12.6 or 15 Gy) in carcinomas, but not in sarcomas [26]. The reduction in ^18^F-FLT PET uptake after two fractions of IMRT (8.4 or 10 Gy) was significant for both tumor types [26]. Visual interpretation of our PET/CT scan results in Figure 1 show a similar tendency as the ^18^F-FLT PET uptake is reduced more than the ^64^Cu-ATSM PET uptake from pre-treatment to mid-treatment, though keeping in mind that our mid-treatment scan is first performed after 22.5 Gy and the ^64^Cu-ATSM scan is performed approximately 6.5 h after injection and not 3 h, as Bradshaw *et al.* used in their study [26]. A recent study of changes in dynamic ^18^F-FDG PET/CT and contrast-enhanced cone beam CT between pre-, mid-, and post-treatment scans during IMRT (40–46 Gy in 10 fractions) of three canine patients with spontaneous head and neck tumors illustrated the attractiveness of doing multi-modal, multi-parameter imaging for evaluating therapy-induced tumor modifications [27]. This study, however, like ours, only included a very limited number of patients. Therefore, further studies are necessary before the incorporation of multiple expensive PET tracers in routine treatment response monitoring might become a reality.

Taken together, our pilot study has presented unique data regarding the simultaneous use of multiple tracers for response monitoring. Using more than one tracer reveals potentially important information about tumor heterogeneity. It is apparent from our study as well as the other studies discussed that different tracers contribute distinctive information about the tumor phenotype, information that may differ between different histopatologic cancer types, as the studies by Bradshaw *et al.* showed [25,26]. Furthermore, being markers of different molecular pathways within the tumor cells and their microenvironment, the individual tracers respond diversely to treatment, as seen in our study. Thus, each tracer adds unique information when using more than one tracer for response monitoring. Further investigations including more canine patients are, however, necessary to elucidate the true value and benefits of using a combination of ^18^F-FDG, ^18^F-FLT, and ^64^Cu-ATSM PET/CT when monitoring response during radiotherapy and to verify the exact significance of using these tracers for individualized treatment planning. Another important aspect to consider is how to interpret the sum of information gained through multi-tracer imaging, and also further study which scan time points are most relevant for response evaluation and prediction of outcome.

Since the biological features of canine cancers resemble human cancers and similar therapies are used for solid tumors in dog and man, further canine studies evaluating multi-tracer PET/CT for therapy planning and response monitoring may give valuable information for both canine and human patients.
